# A Set of 100 Chloroplast DNA Primer Pairs to Study Population Genetics and Phylogeny in Monocotyledons

**DOI:** 10.1371/journal.pone.0019954

**Published:** 2011-05-26

**Authors:** Nora Scarcelli, Adeline Barnaud, Wolf Eiserhardt, Urs A. Treier, Marie Seveno, Amélie d'Anfray, Yves Vigouroux, Jean-Christophe Pintaud

**Affiliations:** 1 UMR Diversité, Adaptation et Développement des Plantes (DIADE), Institut de Recherche pour le Développement (IRD), Montpellier, France; 2 Ecoinformatics and Biodiversity Group, Department of Biological Science, Aarhus University, Aarhus, Denmark; J. Craig Venter Institute, United States of America

## Abstract

Chloroplast DNA sequences are of great interest for population genetics and phylogenetic studies. However, only a small set of markers are commonly used. Most of them have been designed for amplification in a large range of Angiosperms and are located in the Large Single Copy (LSC). Here we developed a new set of 100 primer pairs optimized for amplification in Monocotyledons. Primer pairs amplify coding (exon) and non-coding regions (intron and intergenic spacer). They span the different chloroplast regions: 72 are located in the LSC, 13 in the Small Single Copy (SSC) and 15 in the Inverted Repeat region (IR). Amplification and sequencing were tested in 13 species of Monocotyledons: *Dioscorea abyssinica*, *D. praehensilis*, *D. rotundata*, *D. dumetorum*, *D. bulbifera*, *Trichopus sempervirens* (Dioscoreaceae), *Phoenix canariensis*, *P. dactylifera*, *Astrocaryum scopatum*, *A. murumuru*, *Ceroxylon echinulatum* (Arecaceae), *Digitaria excilis* and *Pennisetum glaucum* (Poaceae). The diversity found in *Dioscorea*, *Digitaria* and *Pennisetum* mainly corresponded to Single Nucleotide Polymorphism (SNP) while the diversity found in Arecaceae also comprises Variable Number Tandem Repeat (VNTR). We observed that the most variable loci (*rps*15-*ycf*1, *rpl*32-*ccs*A, *ndh*F-*rpl*32, *ndh*G-*ndh*I and *ccsA*) are located in the SSC. Through the analysis of the genetic structure of a wild-cultivated species complex in *Dioscorea*, we demonstrated that this new set of primers is of great interest for population genetics and we anticipate that it will also be useful for phylogeny and bar-coding studies.

## Introduction

The knowledge of the chloroplast genome structure and sequence variation in Monocotyledons is still partial and unbalanced. There are currently 25 completely sequenced chloroplast genomes of Monocotyledons available in GenBank [1] but 17 of them are of Poales, and important orders like Liliales, Commelinales and Zingiberales lack complete chloroplast sequences. Comparative genomic analyses of the chloroplast DNA (cpDNA) relevant to Monocotyledons are scarce [2], [3], [4] and mostly focused on grasses and allied groups [5], [6], [7], [8], [9], [10], [11]. A few monocotyledonous species are documented for many genes [12] while numerous species are documented for a few genes only and non-coding regions, including the most commonly used markers for phylogenetic inference and genetic bar-coding like *rbc*L [13], *atp*B [14], *trn*L-F [15], *mat*K, *psb*A-*trn*H, *rpo*C1, *rpo*B-*trn*C, *psb*K-*psb*I, *atp*F-*atp*H, *atp*H-*atp*I [16], [17], [18], [19], [20], [21]. Most other regions of the Large Single Copy (LSC) have been investigated in particular taxa, for example *clp*P intron2 in *Yucca* [22], *rps*2 in *Tiphonium* [23], *pet*N-*psb*M in *Elaeocharis* [24], *psb*D-*trn*T in *Arum* [25], *psb*B and *psb*C in *Vanilla* [26], *psb*Z-*trnf*M in *Livistona* [27], or *acc*D in *Hexalectris* [28], to mention a few studies. Variation within the slowly evolving Inverted Repeat region (IR) [29] has received little attention in Monocotyledons [30], [31], a large part of it being represented only by the complete chloroplast sequences. Within the Small Single Copy (SSC), there is limited information outside the extensively used *ndh*F gene [32], with only few studies using *ycf*1, *rpl*32-*trn*L and *ndh*A [33], [34], [35], [36].

Moreover, available sets of primers for direct sequencing of chloroplast regions in Angiosperms mostly focus on non-coding regions of the LSC [37], [38], [39] while published information on primers for genes is very dispersed [40].

The possibility of screening a large number of loci is useful to detect polymorphic Small Inversions, microsatellites and minisatellites (i.e. Variable Number Tandem Repeats, VNTR) in species complexes and at the population level [1], [37]. Minute and medium size inversions are frequent features of the non-coding cpDNA [41], [42], detectable only through sequencing and showing intraspecific variability [1], [43]. Microsatellites are also widespread structures in non-coding cpDNA that became important population genetics markers [44]. The most common and most widely used microsatellites are mononucleotide repeats [45]. Longer motifs, in particular minisatellites, are comparatively rare, but also proved to be valuable markers [46], [47], [48].

Here we propose a large set of primer pairs optimized for PCR amplification and overlapping sequencing in Monocotyledons. Primers pairs are distributed throughout the whole chloroplast genome and include exons, introns and Intergenic Spacers (IGS) with contrasted mutation rates and evolutionary patterns. They are thus suitable for a wide range of studies from higher-level phylogeny to population genetics. As an example, we used the newly defined primer pairs to study intra-specific cpDNA diversity of three different yam species (*Dioscorea* spp.)

## Materials and Methods

### Primer definition

The complete sequence of six Monocotyledons chloroplast genomes were downloaded from GenBank, namely *Acorus calamus* (NC_007407), *Dioscorea elephantipes* (NC_009601), *Lemna minor* (NC_010109), *Oryza nivara* (NC_005973), *Phalaenopsis aphrodite* (NC_007499) and *Zea mays* (NC_001666).

Segments of these sequences equivalent to two to six genes were aligned using the program Geneious [49]. Consensus primers anchored in exons were designed using Primer3 [50] incorporated in Geneious, in order to amplify IGS, introns or exons. A total of 105 primers pairs were designed, and 100 successfully amplified: 72 in the Large Single Copy region (LSC), 13 in the Small Single Copy (SSC) and 15 in the Inverted Repeat region (IR). Primer sequences, annealing temperature for PCR amplification, and amplification results are summarized in Table S1.

### Test for amplification

Amplification was tested in 13 species of Monocotyledons: 6 Dioscoreaceae species (1 individual each of *Dioscorea abyssinica*, *D. praehensilis*, *D. rotundata*, *D. dumetorum*, *D. bulbifera* and *Trichopus sempervirens*), 5 Arecaceae species (1 individual each of *Phoenix canariensis*, *P. dactylifera*, *Astrocaryum scopatum*, *A. murumuru* and 2 individuals of *Ceroxylon echinulatum*), *Digitaria excilis* (5 individuals) and *Pennisetum glaucum* (6 individuals). Sequences have been deposited in GenBank under accession number JF705257-JF705858, JF745569-JF745769 and JF758190-JF758233.

Amplification was done according to the recommended protocols using either GoTaq (Promega) in its buffer with 5 mM of dNTPs for *D. excilis* and *P. glaucum* or Failsafe enzyme mix (Epicentre) in premix E for Dioscoreaceae and Arecaceae species. Reaction was done in 25 µL with 25 ng of DNA. The initial denaturation (94°C, 3 min) was followed by 35 cycles of denaturation (94°C, 30 s), annealing (Tm, 30 s) and elongation (72°C, 1 min) and by a final elongation step (72°C, 10 min). Amplification was checked on agarose gel.

### Sequencing

PCR products were purified using Ampure (Agencourt) following the recommended protocol. The sequencing PCRs were done using the BigDye terminator kit (Applied Biosystems). PCR products were purified using CleanSeq (Agencourt) and were run on ABI prism 3130 (Applied Biosystems). Note that for *D. excilis* and *P. glaucum*, only a subset of the PCR products was sequenced. Dioscoreaceae and Arecaceae species were sequenced in forward and reverse direction while *D. excilis* and *P. glaucum* were sequenced in forward direction only.

### Data analysis

Sequences were aligned with the program Geneious [49]. Intra-generic diversity was estimated within species or between closely-related species as the number of SNP and the number of Variable Number Tandem Repeats (VNTR). The number of SNP was standardised to 1 kb but length variable parts (e.g., gaps or VNTR) were subtracted from the total length of the alignment. For *D. excilis* and *P. glaucum*, intra-generic diversity was estimated within the analysed species. For Dioscoreaceae, it was estimated between closely-related species (*D. rotundata*, *D. abyssinica* and *D. praehensilis* of subgenus *Eniantophyllum*). For Arecaceae it was estimated as the mean of the diversity found within each species pair in *Phoenix*, *Astrocaryum* and *Ceroxylon*.

Due to high inter-generic divergence in Dioscoreaceae [51] causing alignment difficulties in non-coding regions with *T. sempervirens*, nucleotide diversity was only estimated between two distant species of *Dioscorea*, *D. abyssinica* and *D. elephantipes*. For Arecaceae, an average of three inter-generic comparison, between *Phoenix* (subfamily Coryphoideae) and *Astrocaryum* (subfamilly Arecoideae), *Phoenix* and *Ceroxylon* (subfamily Ceroxyloideae) and *Ceroxylon* and *Astrocaryum* was calculated. Within Poaceae, inter-generic diversity was not estimated for *D. excilis* and *P. glaucum* because only a part of the loci were sequenced. Instead, inter-generic diversity was estimated between *Oryza sativa* and *Zea mays* using the GenBank sequences but restricted to those parts theoretically amplified by the primer pairs tested in the present study.

Comparisons of genetic diversity between SSC, LSC and IR, and between introns, exons and IGS were performed with Kruskal-Wallis tests using the R environment [52], function kruskal.test.

### Example of use for population genetic analysis

We analysed the genetic structure of three yam species (*Dioscorea* spp.) forming a crop-wild relatives complex in Western Africa. The main cultivated yam species in West-Africa is *D. rotundata*. In this region, yam is a staple food but is also culturally extremely important [53]. The wild relatives of *D. rotundata* are *D. abyssinica* and *D. praehensilis* [54], [55]. The three species are genetically different but can hybridize [56].

One sample of each species has been previously sequenced (see above). Based on these sequences, 19 polymorphic loci were identified showing a total of 21 SNP. These 19 loci have been tested on eight additional individuals (four *D. abyssinica* and four *D. praehensilis*) to selected those loci for which polymorphisms were specific to either *D. abyssinica* or *D. praehensilis*; namely *ccs*A-Exon, *ccs*A-*ndh*D, *ndh*H-Exon, *psb*D-Exon and *rrn*4,5-*trn*N.

Finally, a total of 160 *Dioscorea* samples have been amplified using the selected five primers pairs. The sampling included 66 *D. abyssinica*, 39 *D. praehensilis* and 55 *D. rotundata* collected in Benin. A list of individuals and sampling locations is given in the supplementary data file (Table S2). Sequences have been deposited in GenBank under accession number JF757240-JF758189. The five loci revealed six SNP (two for *rrn*4,5-*trn*N and one each for the other loci). A chlorotype is defined as a combination of SNP located on the chloroplast, i.e. a haplotype based on chloroplast SNP. Here, the combinations of the six SNP revealed five chlorotypes. The repartition of chlorotype frequencies among species was compared with a chi-squared test. A MSN, Minimum Spanning Network [57], with chlorotypes was constructed using Haplophyle [58]. MSN illustrates the evolutionary relationships between chlorotypes as a network where the branches represent the differences between sequences data.

## Results and Discussion

### Development of new chloroplast primers

Of the 105 primer pairs designed to sequence the chloroplast genome, 100 amplified consistently and produced good quality sequences. Primers were designed to amplify a wide range of monocotyledons species and we tested them on various species of different genera (*D. abyssinica*, *D. praehensilis*, *D. rotundata*, *D. dumetorum*, *D. bulbifera*, *T. sempervirens*, *P. canariensis*, *P. dactylifera*, *A. scopatum*, *A. murumuru*, *C. echinulatum*, *D. excilis*, *P. glaucum*). Amplification success was 85% (Table S1) which was very similar to the expected mean amplification of 88% derived from the sequences deposited in GenBank used to design the primers (95% for *A. calamus*, 95% for *D. elephantipes*, 97% for *L. minor*, 80% for *O. nivara*, 88% for *P. aphrodite* and 80% for *Z. mays*). Indeed, due to structural changes (inversions, gene loss, etc.) some primers pairs are expected not to amplify in some species. For example, because of the loss of *ycf*2 and *acc*D in *O. nivara* and *Z. mays*, we do not expect amplification with primers pairs *rpl*23-*ycf*2, *ycf*2-*ndh*B, *acc*D-*psa*I, *rbc*L-*acc*D and *acc*D-Exon on these two species.

Primers amplified coding regions (exon 20%), non-coding regions (IGS 35%, intron 9%) and mixed regions (exon+intron 10%, IGS+genes 25%). 75% of these regions were located in the LSC, 12% in the SSC and 15% in the IR.

### Sequence diversity

We obtained a total of 1174 kb sequence data. The analysis covered 78 kb of the chloroplast genome for Dioscoreaceae (51% of the *D. elephantipes* cpDNA), 70 kb for Arecaceae (44% of the *P. dactylifera* cpDNA), 34 kb for *Digitaria* (25% of the *O. nivara* cpDNA) and 20 kb for *Pennisetum* (15% of the *O. nivara* cpDNA).

A summary of intra- and inter-generic diversity results are presented in Table 1. Detailed results are given in supplementary data file (Table S3).

**Table 1:** Observed intra- and inter-generic diversity.

Location	Intra-generic diversity								Inter-generic diversity		
	SNP				VNTR				SNP		
	*Dioscorea*	*Digitaria*	*Pennisetum*	Arecaceae	*Dioscorea*	*Digitaria*	*Pennisetum*	Arecaceae	*Dioscorea*	Poaceae	Arecaceae
LSC	0.18	0	0.34	0.59	0.13	0	0.33	1.02	14.26	62.82	9.07
IR	0.06	0	0	0.16	0	0.13	0.20	0	2.11	11.16	1.44
SSC	0.75	0.31	0	1.07	0.09	0	0	1	23.37	68.84	10.10
Mean	0.24	0.10	0.11	0.57	0.07	0.04	0.18	0.87	13.25	47.61	8.07

Intra-generic diversity was estimated between closely-related species for *Dioscorea* and Arecaceae or within species (*Digitaria excilis* and *Pennisetum glaucum*). Inter-generic diversity was estimated between different genera (Arecaceae and Poaceae) or distant species (*Dioscorea*). The number of SNP was standardised to 1 kb.

### Intra-generic diversity

We found on average a SNP each 1700 bp within the three Arecaceae genera, each 2800 bp between the three *Dioscorea* species *D. abyssinica*, *D. praehensilis* and *D. rotundata*, each 8900 bp among the six *P. glaucum* samples and each 9600 bp among the five *D. excilis* samples. These very low levels of intra-generic diversity in the studied Poaceae suggest a strong bottleneck effects in such cultivated populations. There were few polymorphic microsatellites in *Dioscorea*, *D. excilis* and *P. glaucum*, all mononucleotide, while the Arecaceae exhibit a high number of mono-, di- and 4–8-nucleotide microsatellites as well as minisatellites (Table 2). A total of 66 VNTR were found in palms, 77% of them located in IGS, 23% in intron and none in exon (Table 3). The 51 polymorphic mononucleotide microsatellites encountered within genera and species of palms can be compared with the 342 homopolymers of 7 bp or longer found in the complete chloroplast genome of *Phoenix dactylfiera* [1].

**Table 2:** Comparison of the number and motifs of polymorphic VNTR observed at the intra-generic level.

	*Dioscorea*	*Digitaria*	*Pennisetum*	Arecaceae
n.o. bp sequenced	78134	33862	19746	69422
n.o. mononucleotide	9	0	7	51
n.o. dinucleotide	0	0	0	2
n.o. VNTR with motif >3 bp	0	0	0	13

**Table 3:** Polynucleotide VNTRs with repeat number >2 and polymorphic within genera in palms.

Locus	Motif length	Motif sequence	Number of repeats		
			*Phoenix*	*Ceroxylon*	*Astrocaryum*
trnL intron	2 bp	AT	6	6	8–10
trnL-ndhJ	2 bp	AT	5	6	6–7
trnQ-rps16	4 bp	GATA	3	2	3–4
ndhG-ndhI	5 bp	AAATA	3	3	2–3
trnQ-rps16	6 bp	AATATT	2	2	2–3
rbcL-accD	8 bp	TTACTTAT	1	1	2–3
psbZ-trnfM	12 bp	ACTACTATACTA	2–6	2	3
rpl16-rps3	20 bp	CTCGTTTACAAATATCCAAA	2–3	1	1–2

Interestingly, Arecaceae species exhibit a much higher number of VNTR than *Dioscorea* species. Similar levels of mono- and dinucleotide microsatellites in *Dioscorea* as in closely related palm species could only be found if two distant species (*D. abyssinica* and *D. elephantipes*) were compared (data not shown). This result suggests different evolutionary histories with higher mutation rates and/or larger effective population sizes in Arecaceae than in *Dioscorea* species.

### Inter-generic diversity

Between *O. sativa* and *Z. mays* we found a SNP each 21 bp, between *D. abyssinica* and *D. elephantipes* each 75 bp and for the three inter-generic comparisons in Arecaceae on average each 113 bp. Since *Oryza sativa* and *Zea mays* diverged about 52 MY ago [59] and the compared palm subfamilies diverged about 68–98 MY ago [60], our result confirmed a 5–6 fold faster substitution rate for cpDNA in Poaceae than in Arecaceae [61]. The genus concept in Dioscoreaceae is very different from that of Poaceae and Arecaceae. Levels of divergence between two distant species of *Dioscorea* was in the range of the inter-generic differentiation in Poaceae and Arecaceae, while different Dioscoreaceae genera, namely *Dioscorea* and *Trichopus* are so divergent that they are not even alignable for some IGS.

Interestingly, we did not find significant differences in number of SNP in introns *vs.* exons and in introns *vs.* IGS, neither for *Dioscorea*, nor for Arecaceae and Poaceae (p>0.05 for *Dioscorea*, Arecaceae and Poaceae). We observed a significantly higher number of SNP in IGS *vs.* exons only for *Dioscorea* (p<0.05) and in Poaceae (p<0.01). This finding highlights the very peculiar dynamics of SNP in the chloroplast genome. It can be compared with the result of Yang et al. [1] who identified 62 out of 78 SNP within the cultivar ‘Khalass’ of the date palm occurring in exons, with an unusual synonymous/non synonymous ratio of 0.94. They suggested a lack of purifying selection within heterogeneous intra-individual chloroplast populations as a possible explanation (Yang et al. 2010b).

**Figure 1:**
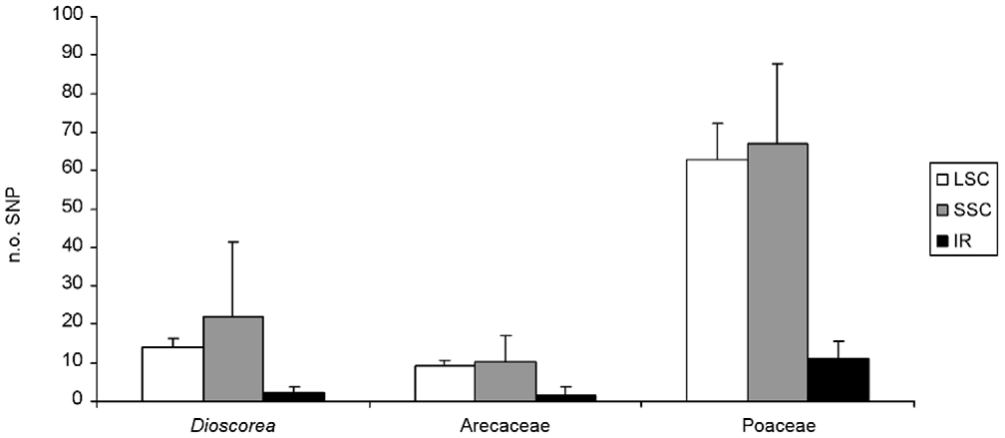
Inter-generic diversity found in *Dioscorea*, Arecaceae and Poaceae. Inter-generic diversity was estimated as the number of SNP in the LSC, SSC and IR. Numbers of SNP have been standardised to 1 kb. Bars represent the 95% confidence intervals.

The occurrence of SNP among the three regions of the chloroplast (LSC, SSC and IR) varies (Figure 1). LSC and SSC exhibit similar levels of diversity while IR exhibits significantly lower numbers of SNP. The difference in number of SNP is significant for LSC *vs.* IR and SSC *vs.* IR (p<0.001 for *Dioscorea*, Arecaceae and Poaceae) but is not significant for LSC vs. SSC (p>0.05 for *Dioscorea*, Arecaceae and Poaceae). Variation in SNP number in the SSC region is, however, mostly driven by the *ndh*F-*rpl*32 locus. This locus exhibits a very high genetic diversity: 92 and 118 SNP per 1 kb for *Dioscorea* and Poaceae, compared to the mean of 16, 10 and 62 SNP per 1 kb for *Dioscorea*, Arecaceae and Poaceae, respectively and of 11, 7 and 30 SNP per 1 kb in the whole chloroplast.

Most published primer pairs focus on non-coding regions of the LSC [37], [38], [39]. This region is commonly used for phylogeny and bar-coding [13], [14], [15], [16], [17], [18], [19], [20]. In the present study, we observed some of the most variable loci in the SSC, namely *rps*15-*ycf*1, *rpl*32-*ccs*A and *ndh*F-*rpl*32 for *Dioscorea* and *rps*15-*ycf*1, *ndh*G-*ndh*I and *ccsA* for Arecaceae.

### Polynucleotide VNTR in palms

Polynucleotide VNTR are apparently rare in Monocotyledons. They are virtually absent in *Dioscorea* and Poaceae, although a 22 bp minisatellite located in the *trn*D-*trn*T region, with 1–3 repeats, has been reported in *Elymus* [47]. A complex evolution of minisatellites was also detected in an orchid, *Anacamptis*, within the *trn*L intron [62].

Palms are outstanding for the frequency of such structures in the chloroplast genome. In this study, 12 VNTR were recorded in the genus *Astrocaryum*, with motif length varying from 2 to 26 bp. There was, however, considerable variation in VNTRs abundance among genera of palms (Table 3). In *Phoenix*, only two polynucleotide VNTRs were detected, namely 2 minisatellites of 12 and 20 bp. Within *C. echinulatum*, there was no polymorphism at the level of the polynucleotide VNTR, and only 9 of the 51 mononucleotide microsatellites were polymorphic. We note, however, that only two individuals have been compared and VNTRs occurrence might be higher. Differences between *Astrocaryum* and *Ceroxylon* might be explained by differences in divergence time between the pairs of individuals compared (less than 2 MY in *Ceroxylon*, about 7 MY in *Astrocaryum*) and also by a higher sequence variability in *Astrocaryum* and other Bactridinae compared with Ceroxyleae and Phoeniceae [63], [64], [65], [66].

Thus, polynucleotide VNTRs have a great potential in palms for population genetic studies and species delimitation. They have already been used with success in several studies. For example, the dodecanucleotide minisatellite of the *psb*Z-*trnf*M locus showed fixed private haplotypes that allowed the separation of closely related *Phoenix* species and tracking interspecific hybridization [48]. The tetranucleotide microsatellite of the *trn*Q-*rps*16 locus allowed tracing seed flow between the wild and cultivated compartments of the peach palm (*Bactris gasipaes*) in western Ecuador and proved to be much more informative than a mononucleotide microsatellite present in the same locus [67].

We note, however, that among the 13 polynucleotide VNTRs with motifs longer than 3 bp found in the palm sampling (Table 2), five are direct repeats, i.e. incipient VNTR with 1–2 units of the motif. Direct repeats are common in non-coding cpDNA [68], and are probably the starting point of more repeated polynucleotide VNTR, although few loci undergo this evolution. For example, a sequence of 8 bp in the *rbc*L-*acc*D spacer was found unrepeated in *Phoenix* and *Ceroxylon* but showed 2–3 tandem repeats in *Astrocaryum* (Table 3). Some minisatellites also originate from inversions [62].

As already noted above, the comparison of a limited number of individuals per family, as in the present study, might considerably underestimates the actual number of VNTR in a given taxa. Indeed, an alignment of the 174 palm sequences deposited in GenBank of the locus *trn*Q-*rps*16 alone (1.1 kb) revealed 16 intra-generic direct repeat polymorphisms 5–22 bp long, a mononucleotide microsatellite with 8–17 repeats, a dinucleotide microsatellite with 3–6 repeats, a tetranucleotide microsatellite with 2–6 repeats and a 26 bp minisatellite with 1–4 repeats. The last structure is polymorphic in a single group, the subtribe Linospadicinae, restricted to the south-west Pacific [69].

For detailed studies of VNTR variation in a particular group, it is therefore advisable to begin with the sequencing of a significant number of samples, in order to evaluate accurately the existing polymorphism in the target locus.

### Example of use for population genetic analysis

CpDNA is generally inherited by only one parent (usually the mother in angiosperms). It is haploid and it generally lacks recombination [70]. CpDNA is therefore of great interest for population genetics studies, including parentage analysis, hybridization, population structure and phylogeography [44].

Here we used the new primer set to study the genetic structure of a yam crop-wild relatives' complex (cultivated: *D. rotundata*, wild: *D. abyssinica* and *D. praehensilis*) in Benin, Western Africa. After screening more than half of the chloroplast genome the set of informative loci retained to study this species complex included four (out of five) loci from the SSC and IR regions, among which three were exons. This emphasizes again how interesting the rarely studied SSC and IR regions are and confirms that exons are not less variable than introns or IGS, as far as it concerns SNP.

**Figure 2:**
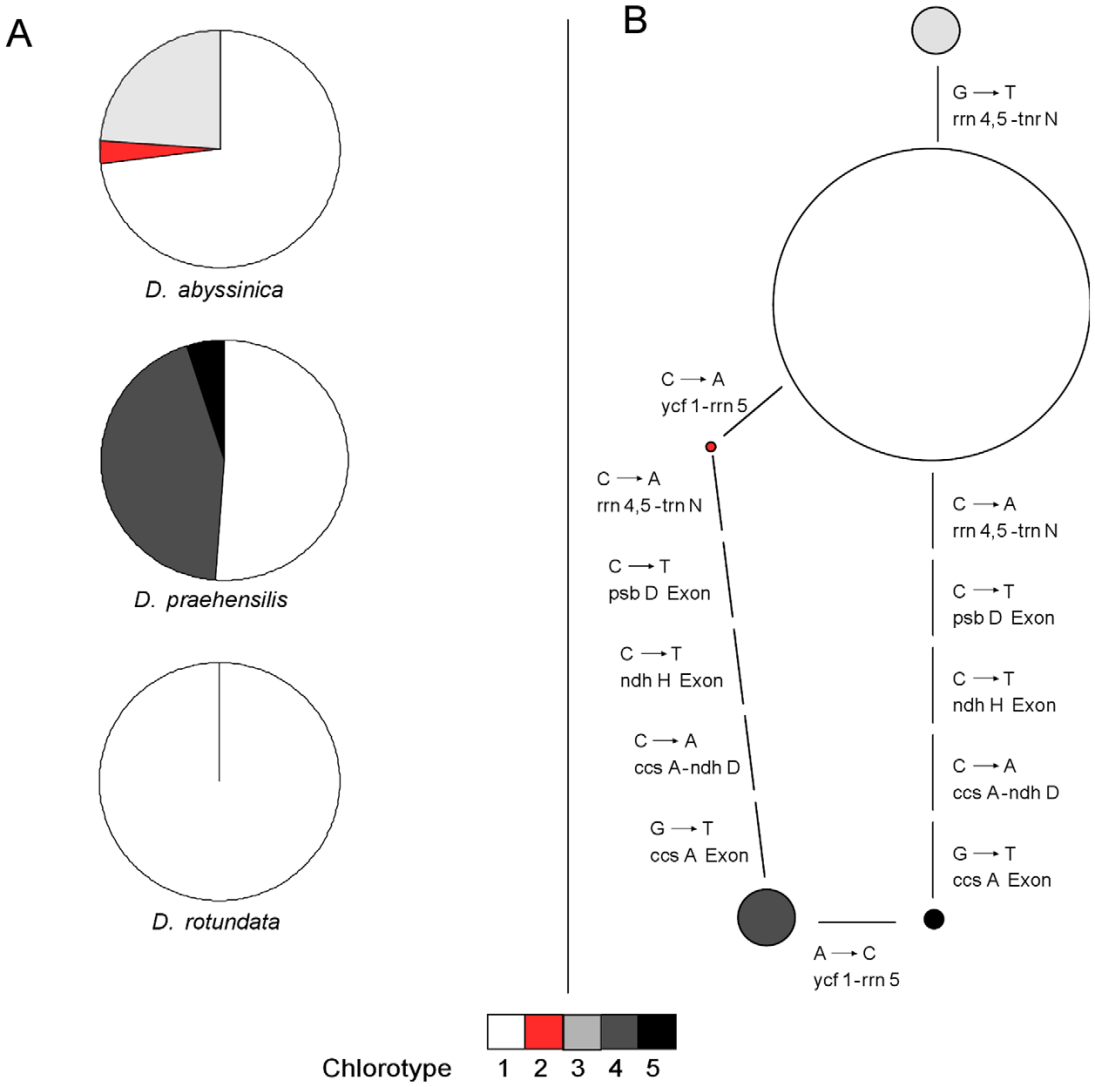
Chlorotypes observed within *Dioscorea* species. (A) Distribution of observed chlorotypes and (B) minimum spanning network (MSN) representing the relationship among chlorotypes. In (B), the size of the circle is proportional to the chlorotype occurrence and each line represents a SNP between the two connected chlorotypes. Each SNP has been labelled with the name of the primer pairs needed for its amplification and the corresponding base change.

We found five chlorotypes among the 160 sequenced *Dioscorea* individuals that showed significantly different frequencies among the three species (Fig. 2a, p<0.001 for all pairwise comparisons, chi-squared tests). Chlorotypes 2 and 3 were specific to *D. abyssinica*; chlorotypes 4 and 5 are specific to *D. praehensilis*; while the most common chlorotype 1 was found in all three species. Chlorotypes 1, 2 and 3 as well as chlorotypes 4 and 5 were closely related with only one SNP separating them (Fig. 2b).

The cultivated species *D. rotundata* harboured only chlorotype 1 and thus was less diverse than its wild relatives *D. abyssinica* and *D. praehensilis*. However, because chlorotype 1 was shared by all three species, we cannot conclude on the maternal origin of *D. rotundata*.

Our results showed that SNP revealed by sequencing can successfully be used to study the diversity of the crop-wild relatives' complex of *Dioscorea*. Furthermore, the genetic diversity revealed by sequencing with five primer pairs was more informative than the genetic diversity observed using five universal chloroplast mononucleotide microsatellites [71].

We thus showed that the new primer set can reveal diversity even when microsatellites might not show polymorphism, as it was the case in the *Dioscorea* species complex studied. We anticipate that the use of sequencing and SNP genotyping for population genetic analysis will be even more interesting for species or species complexes showing higher genetic diversity, as in some groups of Arecaceae like Bactridinae.

### Conclusion

In this paper, we present a large set of newly developed chloroplast DNA primer pairs. Compared to the previously published primer pairs [37], [38], [39], [40], this new set covers a wider range of the chloroplast genome (e.g. up to 51% of the *Dioscorea* cpDNA) and has been designed to optimally amplify in Monocotyledons. This new set of primer pairs spans the Large Single Copy as well as the Small Single Copy and the Inverted Repeats, and has been designed to amplify both coding (exon) and non-coding (intron, intergenic spacer) regions. This new set could be of great interest for phylogeny and bar-coding studies but also for population genetics studies.

## Supporting Information

Table S1 **Primer sequences and amplification range.** Primers were designed using genes alignment of *Dioscorea elephantipes*, *Zea mays*, *Oryza nivara*, *Lemna minor*, *Acorus calamus* and *Phalaenopsis aphrodite*. Amplifications were tested on different species of Dioscoreaceae, *Digitaria*, *Pennisetum* and Arecaceae.
(DOC)

Table S2 **List of** ***Dioscorea*** **individuals used to test the use of the new primers pairs for population genetics studies.** Table includes the chlorplotype (1 to 5) corresponding to each sample.
(DOC)

Table S3 **Observed Intra- and inter-generic diversity.** Intra-generic diversity was estimated between closely-related species for *Dioscorea* and Arecaceae or within species (*Digitaria excilis* and *Pennisetum glaucum*). Inter-generic diversity was estimated between different genera (Arecaceae and Poaceae) or distant species (*Dioscorea*). The number of SNP was standardised to 1 kb.
(DOC)
