# Supplementary material for: A Set of 100 Chloroplast DNA Primer Pairs to Study Population Genetics and Phylogeny in Monocotyledons
Source: PLoS One. 2011 May 26;6(5):e19954. doi: 10.1371/journal.pone.0019954 (PMC3102674; doi:10.1371/journal.pone.0019954)
Supplement: Table S1 — Primer sequences and amplification range. Primers were designed using genes alignment of Dioscorea elephantipes, Zea mays, Oryza nivara, Lemna minor, Acorus calamus and Phalaenopsis aphrodite. Amplifications were tested on different species of Dioscoreaceae, Digitaria, Pennisetum and Arecaceae. (DOC) [file pone.0019954.s001.doc]

**Supplementary data**

Scarcelli et al.

A set of 100 chloroplast DNA primer pairs to study population genetics and phylogeny in Monocotyledons

Table S1. Primer sequences and amplification range.

Primers were designed using genes alignment of *Dioscorea elephantipes*, *Zea mays*, *Oryza nivara*, *Lemna minor*, *Acorus calamus* and *Phalaenopsis aphrodite*. Amplifications were tested on different species of Dioscoreaceae, *Digitaria*, *Pennisetum* and Arecaceae.

Ok = good amplification; - = poor or no amplification; NT = not tested.

| Name | Location | Type | Tm | Forward sequence | Reverse sequence | PCR amplification | | | |
| --- | --- | --- | --- | --- | --- | --- | --- | --- | --- |
|  |  |  |  |  |  | Dioscoreaceae | *Digitaria* | *Pennisetum* | Arecaceae |
| *trn*H-*psb*A | LSC | IGS | 64 | CCACTGCCTTRATCCACTTG | TRGCTGCTTGGCCTGTAGT | - | ok | ok | ok |
| *psb*A Exon | LSC | Exon | 60 | AAGTTGTGWGCATTACGTTC | GCGAAAGTACAAGCCTRTGG | ok | ok | ok | ok |
| *psb*A-*trn*K | LSC | IGS | 62 | GGCTTGTACTTTCGCGTCTC | ATCCGAYTAGTTCCGGGTTC | ok | NT | NT | ok |
| *trn*K-*rps*16 | LSC | IGS | 62 | CTCTACCRTTGAGTTAGCAACC | TGCAATTGATGTTCGATCYC | - | ok | ok | ok |
| *mat*K Exon | LSC | Exon | 53 | RAKAATATCCAAATACCAAA | ACCATATYGYACTATGTATC | ok | ok | ok | ok |
| *rps*16-*trn*Q | LSC | IGS | 62 | GTCGCACGTTGCTTTCTACC | GAGGTTCGAATCCTTYCGTC | - | ok | ok | ok |
| *rps*16 Intron | LSC | Intron | 58 | ATCGAACATCAATTGCAACG | TAGAAAGCAACGTGCGACTT | - | ok | ok | ok |
| *trn*Q-*psb*K | LSC | IGS | 60 | CTGGGACGRAAGGATTCG | ACTTACAGCRGCTTGCCAAA | ok | ok | ok | ok |
| *psb*K-*trn*S | LSC | IGS + Gene | 62 | TTTGGCAAGCYGCTGTAAGT | GGAGAGATGGCTGAGTGGA | ok | ok | ok | ok |
| *trn*S-*trn*G | LSC | IGS | 58 | TCCACTCAGCCATCTCTCC | CRCTTTTACCACTAAACTATACCC | - | ok | ok | - |
| *trn*G Intron | LSC | Intron | 62 | GCGGGTATAGTTTAGTGGTAA | GCTTGGAAGGCTAGGGGTTA | ok | ok | ok | ok |
| *trn*G-*atp*A | LSC | IGS + Gene | 60 | TAACCCCTAGCCTTCCAAGC | TTTGCACAATTYGCTTCTGA | ok | - | - | ok |
| *atp*A Exon | LSC | Exon | 60 | GCCAATTGATTYTGAGTAGCTTT | TGCTCGTRTTCATGGTCTTG | ok | - | - | ok |
| *atp*F-*atp*H | LSC | IGS | 62 | AACTCGCACACACTCCCTTT | GGRGTTGGTCAAGGTACTGC | ok | NT | NT | ok |
| *atp*F Intron/Exon | LSC | Intron + Exon | 56 | AGTWCCTAGAGCTCSTTGT | CCGATATTTTAGCAACAAATC | - | ok | ok | ok |
| atpH-*atp*I | LSC | IGS | 62 | CCAGCAGCAATAACRGAAGC | TTCAAGCTCTTATTTTTGCAACKT | ok | NT | NT | ok |
| *atp*I Exon | LSC | Exon | 56 | ACCYTCCATGGATTCRCC | CAARTYCATGCCCAAGT | ok | ok | ok | ok |
| *atp*I-*rps*2 | LSC | IGS | 56 | AKTTTCCAAGGTAAAAGAGC | CGTAAAGGTAYTCATATTACAAATCT | ok | ok | ok | ok |
| *rps*2 Exon | LSC | Exon | 53 | ACTAATTTGTTAAGAATYAATCG | AAGATATTGGAACATCAATTT | ok | ok | ok | ok |
| *rps*2-*rpo*C2 | LSC | IGS | 56 | TTTGTAATATGARTACCTTTACG | CGRGCAATCTTATTGGGAAT | ok | ok | ok | ok |
| *rpo*C2-*rpo*C1 | LSC | IGS | 60 | CCGAARTGATCTATTAATCTGCT | GATGGRGATCAAATGGCTGT | ok | ok | ok | ok |
| *rpo*C1 Intron/Exon1 | LSC | Intron + Exon | 56 | TCGAATRAACCYCGTAATCG | GTTTCYCCTCAACAAATMA | ok | ok | ok | ok |
| *rpo*C1 Exon2 | LSC | Exon | 54 | TTTCTCKATAAAAAGAAATATGACC | ACGATTACGRGGTTYATTCG | ok | ok | ok | - |
| *rpo*B-*trn*C | LSC | IGS | 58 | GTGAGTGATTCATAYACAGCA | GGCCATGYCGCCAAA | ok | ok | ok | ok |
| *trn*C-*pet*N | LSC | IGS | 58 | GGGGACTGCAAATCCTTT | CATTAAAGCAGCCCAAG | ok | ok | ok | ok |
| *pet*N-*trn*D | LSC | IGS + Gene | 58 | CTTGGGCTGCTTTAATGGT | CTGTCAAGGCGGAAGCTG | ok | ok | ok | - |
| *trn*D-*trn*T | LSC | IGS + Gene | 58 | GGTGCTCTGACCRATTGAACT | GCCCYTTATCGGATTTGAAC | ok | - | - | ok |
| *trn*T-*psb*D | LSC | IGS | 60 | GCCATGGTAAGGCGTAAGTC | CAACCYACAAAAACGAAACG | ok | - | ok | ok |
| *psb*D Exon | LSC | Exon | 60 | CCTTGTGCYTATTTCGCYTTA | TTCATGAGGCTGATCCTGAG | ok | ok | ok | ok |
| *psb*C-*psb*Z | LSC | IGS + Gene | 58 | CAACCTTGGCAAGAACG | TTGACCAACCATCAGRAGA | ok | ok | ok | ok |
| *psb*C Exon | LSC | Exon | 58 | GTGGAAACGCTCTTTAATGG | GCCACAAATGDCCCACAA | ok | ok | ok | ok |
| *psb*Z-*trnf*M | LSC | IGS + Gene | 62 | GGTACMTCATTATGGATTGG | GCGGAGTAGAGCAGTTTGGT | ok | - | - | ok |
| *trnf*M-*psa*B | LSC | IGS + Gene | 62 | GCGAGCTACCAAACTGCTCT | ATAAGCCAGTGGCYCTTTCC | ok | - | ok | ok |
| *psa*A-*ycf*3 | LSC | IGS | 60 | AGCATCRGCATGTAGGTTCC | TCARGCTGCTGAGTATTGGA | ok | ok | ok | ok |
| *ycf*3 Intron2 | LSC | Intron | 60 | GCYTGTTTCCAATACTCAGCA | ATGGCCGTGATCTGTCATTA | ok | ok | ok | ok |
| *ycf*3 Intron1 | LSC | Intron + Exon | 58 | TGACAGATCACGGCCATATT | TTAYAGAGATGGTGCGATTT | ok | ok | ok | ok |
| *ycf*3-rps4 | LSC | IGS + Gene | 58 | TCAAATCGCACCATCTCTRT | AGACAYATTTTAGTTAATGGTCGT | ok | ok | ok | ok |
| *trn*L Intron | LSC | Intron | 62 | GGTAGACGCTACGGACTT | TGGGGATAGAGGGACTTGAA | ok | ok | ok | ok |
| *trn*L-*ndh*J | LSC | IGS + Gene | 58 | ARTCGTGAGGGTTCAAGTCC | ATGCCYGAAAGTTGGATAGG | ok | ok | ok | - |
| *ndh*C-*trn*V | LSC | IGS | 54 | CGCCATGCATAAACTRAACC | AATGCATGTTGGGTCTTTGA | ok | ok | ok | - |
| *trn*V Intron | LSC | Intron | 62 | GAACCGTAGACCTTCTCGGTAA | GTTTACACGYGCGCCAAT | ok | ok | ok | ok |
| *trn*V-*atp*B | LSC | IGS + Gene | 62 | CGTGTAAACGAGKTGCTCTACC | GGGKAACATCGATGAAGCTA | ok | ok | ok | ok |
| *atp*B Exon | LSC | Exon | 60 | TCATTTCTTCAATTTGYTCTCC | GAGCTGTRGCTATGAGTGCT | ok | ok | ok | ok |
| *atp*B-*rbc*L | LSC | IGS | 64 | AGCACTCATAGCYACAGCTC | TGCTTTAGTYTCTGTTTGTGGTG | ok | ok | ok | ok |
| *rbc*L Exon | LSC | Exon | 58 | TGYTGGATTYAAAGCTGGTGT | TCRTTACGAGCTTKTACACA | ok | ok | ok | ok |
| *rbc*L-*acc*D | LSC | IGS | 56 | GCTTCWGGKGGTATTCATGT | YATTGTCAATMTCAAAAATCTG | ok | - | - | ok |
| *acc*D-*psa*I | LSC | IGS | 54 | AATGCCTACATTGCRTTTGC | TGCCGGAAAKACTAGGC | ok | - | - | - |
| *acc*D exon | LSC | Exon | 53 | TCAGATTTTTGAKATTGACA | GCAAAYGCAATGTAGGCAT | ok | - | - | ok |
| *psa*I-*ycf*4 | LSC | IGS | 56 | TTTCCGGCAWTYGCAAT | TGTTCTGATCGCCARTTCAT | ok | ok | ok | ok |
| *ycf*4 Exon | LSC | Exon | 58 | ATGAAYTGGCGATCAGAACA | TCAGTACGRGTCAAGGGAAT | ok | ok | ok | ok |
| *ycf*4-*pet*A | LSC | IGS | 56 | ATGAAYTGGCGATCAGAACA | TGYGCAAAAATGGGATATG | - | ok | ok | ok |
| *pet*A-*psb*L | LSC | IGS + Gene | 58 | TTTGAAAAGGTTCARTTGT | AATGTTGAATTRAATCGTACC | - | - | ok | ok |
| *pet*A Exon | LSC | Exon | 56 | AYGCATATCCCATTTTTGC | TCATTTCGWACAAYTGAACC | ok | ok | ok | ok |
| *pet*L-*trn*P | LSC | IGS + Gene | 60 | TGGCTGCTTYAACTMTARCC | CTTGGTAGCGCGTTTGTTTT | ok | ok | ok | ok |
| *trn*P-*rps*18 | LSC | IGS + Gene | 58 | AAAACAAACGCGCTACCAAG | AAATCGAYTAATTAAACTCATGTTTC | ok | ok | ok | ok |
| *rps*18-*rps*12 | LSC | IGS + Gene | 56 | ACYTTGAAACAACAACGATTA | TCGAGGAACATGTRCTAGGG | ok | ok | ok | ok |
| *rps*12-*clp*P | LSC | IGS | 60 | ACATGTTCCTCGACGCTGA | AAACGYCTAGCATTCCCTCA | ok | NT | NT | ok |
| *clp*P Intron2 | LSC | Intron | 60 | AAAAGAACTRGCAGGTTGGTG | AAACGYCTAGCATTCCCTCA | ok | - | ok | ok |
| *clp*P Intron1 | LSC | Intron + Exon | 64 | GAGGGAATGCTAGRCGTTTG | GGARTCCTGGAGAGGAAGATG | ok | - | - | ok |
| *rps*12-*psb*B | LSC | IGS + Gene | 58* | ACATGTTCCTCGACGCTGA | CGACCSGGATCATTCAATAC | - | ok | ok | - |
| *psb*B Exon | LSC | Exon | 60 | GGGTTTRCCTTGGTATCGTG | TCTGGATCAATACCRGCAAA | ok | ok | ok | ok |
| *clp*P-*psb*B | LSC | IGS | 58 | GAGGGAATGCTAGRCGTTTG | CGACCSGGATCATTCAATAC | ok | NT | NT | ok |
| *pet*B Intron/Exon2 | LSC | Intron + Exon | 60 | TGAGYGTGTGACTTGTTAKAAT | CCYGAAATMCCTTGCTTACG | ok | ok | ok | ok |
| *pet*B-*pet*D | LSC | IGS | 60 | GCAAGGTATTTCRGGTCCTTT | TTATGTCCCATCCCTTTAGC | - | ok | ok | ok |
| *pet*D Intron/Exon2 | LSC | Intron + Exon | 58 | ATGGGAGTGTGYGACTTGAA | CGACGAAAKGGATTTTGGA | ok | ok | ok | ok |
| *pet*D-*rpo*A | LSC | IGS | 56 | AAATTCCAAAATCCMTTTCGTC | AATGGAAGTTTAACYCCTAA | ok | ok | ok | ok |
| *rps*11-*rps*8 | LSC | IGS + Gene | 52 | TGAAACTYGCYTGAACATGAA | TTGAAAACGTTMGAAAACATC | ok | ok | ok | - |
| *rps*8-*rpl*16 | LSC | IGS + Gene | 60 | GGTCGGCTGATACGYT | GAGGAGCYGGATGAGAAGAA | ok | ok | ok | ok |
| *rpl*16 Intron | LSC | Intron | 60 | CCYYTCATTCTTCCTCTATGTTG | TGCTTAGTGTGTGACTCGTT | ok | ok | ok | ok |
| *rpl*16-*rps*3 | LSC | IGS | 62 | AGTCACACACTRAGCATAGCA | TCCACTYGGTTTCAGACTTGG | ok | ok | ok | ok |
| *rps*3 Exon | LSC | Exon | 60 | CACGYGCAATYTCTTTTC | TCCACTYGGTTTCAGACTTGG | ok | ok | ok | ok |
| *rpl*22-*rpl*2 | LSC | IGS + Gene | 58 | TTTGCTGCTGCMGARTAAAC | GAGCCGGRTCTAAGTGTTGG | ok | ok | ok | - |
| *rpl*2 Intron/Exon1-2 | IR | Intron + Exon | 62 | TTACGGCGACGAAGAATMAA | GRCATAGAGGGGGAGGTCAT | ok | ok | ok | ok |
| *rpl*23-*ycf*2 | IR | IGS + Gene | 60 | AGAAGAGTTCGACCCAATGC | AATTCGGGGGTCAAAGAGTT | ok | ok | ok | ok |
| *ycf*2-*ndh*B | IR | IGS + Gene | 54 | CTTTTCCCGGATGAAATGAA | CCAAGAAATAACCCCTCACG | ok | ok | - | - |
| *ndh*B Exon2 | IR | Exon | 58 | TCCTGAGCAATTGCAAGAAT | AAAGTCTCATGCACGGTTTTG | ok | ok | ok | ok |
| *ndh*B Intron/Exon1 | IR | Intron + Exon | 60 | CAAAACCGTGCATGAGACTTT | TGATCTGGCATGTACAGAATGAA | ok | ok | ok | ok |
| *ndh*B-*rps*7 | IR | IGS | 58 | CGTTGAAATTGTTCGTTTGG | ATGGCAGAGGCAAATAGAGC | ok | ok | ok | ok |
| *rps*12-*trn*V | IR | IGS | 58 | GGCAACTTTACGTAAGGCAGAG | CGAACTGATGACTTCCACCA | ok | ok | ok | - |
| *rps*12 Intron/Exon | IR | Intron + Exon | 62 | CTCCCTCCAAGCCGTACATA | GCCTTACGTAAAGTTGCCAGA | ok | ok | ok | ok |
| *trn*V-*rrn*16 | IR | IGS | 64 | ACCTTGACGTGGTGGAAGTC | TGAGCCAGGATCGAACTCTC | ok | ok | ok | ok |
| *rrn*16 Exon | IR | Exon | 64 | GAGAGTTCGATCCTGGCTCA | GCACCTTCCAGTACGGCTAC | ok | ok | ok | ok |
| *rrn*16-*trn*I | IR | IGS | 64 | GGTAGCCGTACTGGAAGGTG | CGCGCTCTACCACTGAGCTA | ok | ok | ok | ok |
| *trn*I Intron | IR | Intron | 64 | TAGAGCGCGCCCCTGATAAT | CCATCCTGGACTTGAACCAG | ok | ok | ok | ok |
| *trn*A-*rrn*23 | IR | IGS | 60 | TTCGAGTCCGCTTATCTCCA | GAAGCATTTCGTCGCTTRCT | ok | ok | ok | ok |
| *trn*A Intron | IR | Intron | 62 | TTGGTAGAGCTCCGCTCTTG | GACTCGAACCGCTGACATC | ok | ok | ok | ok |
| *rrn*4,5-*trn*N | IR | IGS + Gene | 64 | GYCAAGTGGAAGTGCAGTGA | GGTAGAGCGGTYGGCTGTTA | ok | ok | ok | ok |
| *rps*15-*ycf*1 | SSC | IGS | 62 | CGTTGACGTTTTCCYAGRATT | GCTTGTATGAATCGBTATTGGTTT | ok | - | - | ok |
| *ycf*1-*rrn*5 | SSC | IGS + Gene | 62 | ATGCAGAGGCGCATAATAGA | CTGCGGAAAAATAGCTCGAC | - | ok | - | ok |
| *ycf*1-*rrn*5 Dio | SSC | IGS + Gene | 58 | AAAATAGCTCGACGCCAGAA | GGATAATAGATTACCTAGTAGAAAAG | ok | ok | ok | ok |
| *ndh*A Intron | SSC | Intron + Exon | 58 | TCYGCTTCTGGTAAATCAAA | AATATCTCTACGTGYGATTCG | ok | ok | ok | ok |
| *ndh*H Exon | SSC | Exon | 58 | GGACGAATTTTCCATCTCC | CATCAATGCAYGGTGTTCT | ok | ok | ok | ok |
| *ndh*G-*ndh*I | SSC | IGS | 58 | GCCATASTAATTGCACCT | TYGCTTGTGAAGTATGTGTTCG | ok | ok | ok | ok |
| *ndh*G Exon | SSC | Exon | 60 | CGMGCCATASTAATTGCACCT | TTTAMYTGGRCCAATACATGA | ok | ok | - | ok |
| *psa*C-*ndh*G | SSC | IGS + Gene | 54 | TCRCATCTCTTACAACCYACACA | GTTCAACARATTGGRATTCAT | ok | ok | ok | - |
| *ccs*A-*ndh*D | SSC | IGS | 60 | GCAGTRTGGGCTAATGAGG | GGAATGAGYGGTTTTGTTGC | ok | ok | ok | ok |
| *ndh*D Exon | SSC | Exon | 58 | GGAGTTAATATMATTCCWATTGC | TAGCRGCTTGGCCRGTTAC | ok | ok | ok | ok |
| *ccs*A Exon | SSC | Exon | 53 | ACTTTAGAACAYATAYTAACTCA | CCAAAATARCAKATCCAAAT | ok | ok | ok | ok |
| *rpl*32-*ccs*A | SSC | IGS + Gene | 58 | ATGGCRGTYCCAAARAAACG | GCYTCATTAGCCCAYACTGC | ok | ok | ok | - |
| *ndh*F-*rpl*32 | SSC | IGS | 54 | GCATATTGATAKGTATGTTCCA | ATMGAAGTRCGTTTYTTTGG | ok | ok | ok | ok |
| % of amplification | LSC |  |  |  |  | 0.86 | 0.76 | 0.82 | 0.88 |
| % of amplification | IR |  |  |  |  | 1 | 1 | 0.93 | 0.87 |
| % of amplification | SSC |  |  |  |  | 0.92 | 0.92 | 0.77 | 0.85 |
| % of amplification | Total |  |  |  |  | 0.89 | 0.82 | 0.83 | 0.87 |
