# Supplementary material for: A Set of 100 Chloroplast DNA Primer Pairs to Study Population Genetics and Phylogeny in Monocotyledons
Source: PLoS One. 2011 May 26;6(5):e19954. doi: 10.1371/journal.pone.0019954 (PMC3102674; doi:10.1371/journal.pone.0019954)
Supplement: Table S2 — List of Dioscorea individuals used to test the use of the new primers pairs for population genetics studies. Table includes the chlorplotype (1 to 5) corresponding to each sample. (DOC) [file pone.0019954.s002.doc]

**Supplementary data**

Scarcelli et al.

A set of 100 chloroplast DNA primer pairs to study population genetics and phylogeny in Monocotyledons

Table S2. List of *Dioscorea* individuals used to test the use of the new primers pairs for population genetics studies. Table includes the chlorplotype (1 to 5) corresponding to each sample.

| Individual | Species | Longitude | Latitude | Chlorotype |
| --- | --- | --- | --- | --- |
|
| A227 | *D. abyssinica* | 2.29 | 9.79 | 1 |
| A231 | *D. abyssinica* | 2.71 | 9.82 | 1 |
| A238 | *D. abyssinica* | 2.3 | 10.34 | 1 |
| A242 | *D. abyssinica* | 2.42 | 10.24 | 1 |
| A245 | *D. abyssinica* | 2.43 | 10.24 | 3 |
| A249 | *D. abyssinica* | 2.53 | 10.48 | 1 |
| A250 | *D. abyssinica* | 2.53 | 10.51 | 1 |
| A251 | *D. abyssinica* | 2.43 | 10.24 | 1 |
| A259 | *D. abyssinica* | 2.5 | 10.48 | 1 |
| A262 | *D. abyssinica* | 2.23 | 10.34 | 1 |
| A263 | *D. abyssinica* | 2.23 | 10.34 | 3 |
| A264 | *D. abyssinica* | 2.41 | 10.23 | 1 |
| A269 | *D. abyssinica* | 1.51 | 11.12 | 1 |
| A270 | *D. abyssinica* | 1.53 | 11.22 | 1 |
| A273 | *D. abyssinica* | 1.3 | 10.91 | 1 |
| A275 | *D. abyssinica* | 1.91 | 11.41 | 1 |
| A292 | *D. abyssinica* | 2.38 | 8.5 | 1 |
| A298 | *D. abyssinica* | 2.62 | 8.68 | 1 |
| A299 | *D. abyssinica* | 2.65 | 8.69 | 1 |
| A302 | *D. abyssinica* | 1.98 | 8.37 | 1 |
| A303 | *D. abyssinica* | 1.98 | 8.37 | 1 |
| A305 | *D. abyssinica* | 1.98 | 8.37 | 1 |
| A308 | *D. abyssinica* | 1.98 | 8.37 | 1 |
| A414 | *D. praehensilis* | 1.94 | 7.34 | 4 |
| A415 | *D. praehensilis* | 1.94 | 7.34 | 4 |
| A421 | *D. praehensilis* | 1.8 | 7.52 | 1 |
| A442 | *D. abyssinica* | 2.5 | 8.91 | 1 |
| A465 | *D. abyssinica* | 2.16 | 10.23 | 1 |
| A466 | *D. abyssinica* | 2.32 | 10.23 | 3 |
| A467 | *D. abyssinica* | 2.32 | 10.23 | 1 |
| A468 | *D. abyssinica* | 2.32 | 10.23 | 1 |
| A478 | *D. abyssinica* | 2.32 | 10.23 | 1 |
| A480 | *D. abyssinica* | 2.32 | 10.23 | 1 |
| A483 | *D. abyssinica* | 2.32 | 10.22 | 2 |
| A486 | *D. abyssinica* | 2.32 | 10.22 | 2 |
| A487 | *D. abyssinica* | 2.32 | 10.22 | 1 |
| A494 | *D. abyssinica* | 2.33 | 10.23 | 3 |
| A503 | *D. abyssinica* | 2.31 | 10.19 | 3 |
| A507 | *D. abyssinica* | 2.31 | 10.19 | 3 |
| A517 | *D. abyssinica* | 2.31 | 10.19 | 1 |
| A520 | *D. abyssinica* | 2.31 | 10.19 | 1 |
| A540 | *D. abyssinica* | 2.31 | 10.18 | 1 |
| A546 | *D. abyssinica* | 2.31 | 10.18 | 1 |
| A551 | *D. abyssinica* | 2.3 | 10.2 | 1 |
| A552 | *D. abyssinica* | 2.3 | 10.2 | 3 |
| A554 | *D. abyssinica* | 2.3 | 10.2 | 3 |
| A557 | *D. abyssinica* | 2.3 | 10.2 | 3 |
| A559 | *D. abyssinica* | 2.3 | 10.2 | 3 |
| A561 | *D. abyssinica* | 2.3 | 10.2 | 1 |
| A565 | *D. abyssinica* | 2.31 | 10.22 | 1 |
| A569 | *D. abyssinica* | 2.31 | 10.22 | 1 |
| A571 | *D. abyssinica* | 2.31 | 10.22 | 1 |
| A573 | *D. abyssinica* | 2.31 | 10.21 | 3 |
| A575 | *D. abyssinica* | 2.31 | 10.21 | 3 |
| A577 | *D. abyssinica* | 2.31 | 10.22 | 3 |
| A582 | *D. abyssinica* | 2.31 | 10.21 | 3 |
| A585 | *D. abyssinica* | 2.31 | 10.21 | 1 |
| A587 | *D. abyssinica* | 2.31 | 10.21 | 1 |
| A589 | *D. abyssinica* | 2.31 | 10.21 | 1 |
| A595 | *D. abyssinica* | 2.31 | 10.21 | 3 |
| A598 | *D. abyssinica* | 2.31 | 10.21 | 1 |
| A617 | *D. praehensilis* | 1.1 | 7.7 | 1 |
| A622 | *D. praehensilis* | 1.8 | 7.53 | 1 |
| A623 | *D. praehensilis* | 1.8 | 7.53 | 1 |
| A70 | *D. abyssinica* | 2.05 | 8.44 | 1 |
| A83 | *D. abyssinica* | 1.92 | 10.25 | 1 |
| A85 | *D. abyssinica* | 2.7 | 9.77 | 1 |
| A89 | *D. abyssinica* | 1.98 | 8.37 | 1 |
| CR103 | *D. rotundata* | 2.27 | 10.4 | 1 |
| CR113 | *D. rotundata* | 2.05 | 8.44 | 1 |
| CR114 | *D. rotundata* | 1.97 | 8.37 | 1 |
| CR115 | *D. rotundata* | 1.97 | 8.37 | 1 |
| CR117 | *D. rotundata* | 1.8 | 7.52 | 1 |
| CR118 | *D. rotundata* | 1.74 | 7.24 | 1 |
| CR137 | *D. rotundata* | 1.8 | 7.52 | 1 |
| CR140 | *D. rotundata* | 1.74 | 7.24 | 1 |
| CR147 | *D. rotundata* | 1.74 | 7.24 | 1 |
| CR150 | *D. rotundata* | 1.74 | 7.24 | 1 |
| CR160 | *D. rotundata* | 1.8 | 7.52 | 1 |
| CR166 | *D. rotundata* | 2.05 | 8.44 | 1 |
| CR168 | *D. rotundata* | 2.05 | 8.44 | 1 |
| CR170 | *D. rotundata* | 2.1 | 7.5 | 1 |
| CR19 | *D. rotundata* | 2.05 | 8.44 | 1 |
| CR20 | *D. rotundata* | 1.97 | 8.37 | 1 |
| CR21 | *D. rotundata* | 1.97 | 8.37 | 1 |
| CR22 | *D. rotundata* | 2.05 | 8.44 | 1 |
| CR23 | *D. rotundata* | 2.05 | 8.44 | 1 |
| CR24 | *D. rotundata* | 1.97 | 8.37 | 1 |
| CR26 | *D. rotundata* | 1.97 | 8.37 | 1 |
| CR628 | *D. rotundata* | 2.3 | 10.2 | 1 |
| CR629 | *D. rotundata* | 2.3 | 10.2 | 1 |
| CR630 | *D. rotundata* | 2.3 | 10.2 | 1 |
| CR634 | *D. rotundata* | 2.3 | 10.2 | 1 |
| CR638 | *D. rotundata* | 2.3 | 10.2 | 1 |
| CR641 | *D. rotundata* | 2.3 | 10.2 | 1 |
| CR642 | *D. rotundata* | 2.3 | 10.2 | 1 |
| CR644 | *D. rotundata* | 2.3 | 10.2 | 1 |
| CR647 | *D. rotundata* | 2.3 | 10.2 | 1 |
| CR648 | *D. rotundata* | 2.3 | 10.2 | 1 |
| CR649 | *D. rotundata* | 2.3 | 10.2 | 1 |
| CR651 | *D. rotundata* | 2.3 | 10.2 | 1 |
| CR660 | *D. rotundata* | 2.3 | 10.2 | 1 |
| CR665 | *D. rotundata* | 2.3 | 10.2 | 1 |
| CR694 | *D. rotundata* | 2.42 | 10.24 | 1 |
| CR701 | *D. rotundata* | 1.8 | 7.53 | 1 |
| CR703 | *D. rotundata* | 1.8 | 7.53 | 1 |
| CR704 | *D. rotundata* | 1.8 | 7.53 | 1 |
| CR76 | *D. rotundata* | 2.27 | 10.4 | 1 |
| CR829 | *D. rotundata* | 2.05 | 8.44 | 1 |
| CR830 | *D. rotundata* | 2.05 | 8.44 | 1 |
| CR831 | *D. rotundata* | 2.05 | 8.44 | 1 |
| CR833 | *D. rotundata* | 1.97 | 8.37 | 1 |
| CR835 | *D. rotundata* | 1.97 | 8.37 | 1 |
| CR836 | *D. rotundata* | 1.97 | 8.37 | 1 |
| CR838 | *D. rotundata* | 1.74 | 7.24 | 1 |
| CR840 | *D. rotundata* | 1.74 | 7.42 | 1 |
| CR844 | *D. rotundata* | 1.75 | 7.43 | 1 |
| CR845 | *D. rotundata* | 1.74 | 7.24 | 1 |
| CR846 | *D. rotundata* | 1.74 | 7.24 | 1 |
| CR850 | *D. rotundata* | 1.8 | 7.52 | 1 |
| CR852 | *D. rotundata* | 1.8 | 7.52 | 1 |
| CR90 | *D. rotundata* | 2.27 | 10.4 | 1 |
| CR98 | *D. rotundata* | 1.79 | 7.4 | 1 |
| P235 | *D. praehensilis* | 1.7 | 9.67 | 1 |
| P282 | *D. praehensilis* | 1.91 | 8.17 | 4 |
| P283 | *D. praehensilis* | 1.91 | 8.17 | 4 |
| P286 | *D. praehensilis* | 1.85 | 8.32 | 4 |
| P297 | *D. praehensilis* | 2.46 | 7.76 | 4 |
| P397 | *D. praehensilis* | 1.64 | 7.34 | 1 |
| P402 | *D. praehensilis* | 1.64 | 7.34 | 1 |
| P404 | *D. praehensilis* | 1.64 | 7.34 | 1 |
| P405 | *D. praehensilis* | 1.64 | 7.34 | 1 |
| P407 | *D. praehensilis* | 1.64 | 7.34 | 1 |
| P409 | *D. praehensilis* | 1.64 | 7.34 | 1 |
| P42 | *D. praehensilis* | 1.52 | 9.25 | 1 |
| P427 | *D. praehensilis* | 1.82 | 7.5 | 4 |
| P428 | *D. praehensilis* | 1.82 | 7.5 | 4 |
| P429 | *D. praehensilis* | 1.82 | 7.5 | 4 |
| P439 | *D. praehensilis* | 1.72 | 7.42 | 4 |
| P445 | *D. praehensilis* | 2.24 | 6.71 | 4 |
| P446 | *D. praehensilis* | 2.24 | 6.71 | 4 |
| P447 | *D. praehensilis* | 2.24 | 6.71 | 4 |
| P456 | *D. praehensilis* | 2.16 | 6.39 | 4 |
| P457 | *D. praehensilis* | 2.16 | 6.39 | 4 |
| P458 | *D. praehensilis* | 2.16 | 6.39 | 4 |
| P460 | *D. praehensilis* | 2.13 | 6.39 | 5 |
| P462 | *D. praehensilis* | 2.16 | 6.39 | 5 |
| P599 | *D. praehensilis* | 1.82 | 7.5 | 1 |
| P601 | *D. praehensilis* | 1.82 | 7.49 | 1 |
| P602 | *D. praehensilis* | 1.82 | 7.49 | 1 |
| P603 | *D. praehensilis* | 1.82 | 7.49 | 1 |
| P606 | *D. praehensilis* | 1.82 | 7.5 | 4 |
| P607 | *D. praehensilis* | 1.82 | 7.49 | 1 |
| P609 | *D. praehensilis* | 1.79 | 7.39 | 1 |
| P611 | *D. praehensilis* | 1.79 | 7.39 | 1 |
| P614 | *D. praehensilis* | 1.79 | 7.39 | 1 |
| P624 | *D. praehensilis* | 1.8 | 7.53 | 1 |
| P78 | *D. praehensilis* | 1.52 | 9.25 | 4 |
| P86 | *D. praehensilis* | 2.43 | 7.48 | 4 |
| P87 | *D. praehensilis* | 1.64 | 7.34 | 1 |
