# Supplementary material for: A Set of 100 Chloroplast DNA Primer Pairs to Study Population Genetics and Phylogeny in Monocotyledons
Source: PLoS One. 2011 May 26;6(5):e19954. doi: 10.1371/journal.pone.0019954 (PMC3102674; doi:10.1371/journal.pone.0019954)
Supplement: Table S3 — Observed Intra- and inter-generic diversity. Intra-generic diversity was estimated between closely-related species for Dioscorea and Arecaceae or within species (Digitaria excilis and Pennisetum glaucum). Inter-generic diversity was estimated between different genera (Arecaceae and Poaceae) or distant species (Dioscorea). The number of SNP was standardised to 1 kb. (DOC) [file pone.0019954.s003.doc]

**Supplementary data**

Scarcelli et al.

A set of 100 chloroplast DNA primer pairs to study population genetics and phylogeny in Monocotyledons

Table S3. Observed Intra- and inter-generic diversity. Intra-generic diversity was estimated between closely-related species for *Dioscorea* and Arecaceae or within species (*Digitaria excilis* and *Pennisetum glaucum*). Inter-generic diversity was estimated between different genera (Arecaceae and Poaceae) or distant species (*Dioscorea*). The number of SNP was standardised to 1kb.

- = no amplification; NT = Not tested

| Fragment name | Location | Type | Intra-generic diversity | | | | | | | | Inter-generic diversity | | |
| --- | --- | --- | --- | --- | --- | --- | --- | --- | --- | --- | --- | --- | --- |
|  |  |  | SNP | | | | VNTR | | | | SNP | | |
|  |  |  | *Dioscorea* | *Digitaria* | *Pennisetum* | Arecaceae | *Dioscorea* | *Digitaria* | *Pennisetum* | Arecaceae | *Dioscorea* | Poaceae | Arecaceae |
| *trn*H-*psb*A | LSC | IGS | - | 0 | NT | 1.03 | - | 0 | NT | 0 | - | 45.63 | 0 |
| *psb*A Exon | LSC | Exon | 0 | NT | NT | 0.37 | 0 | NT | NT | 0 | 10.14 | 32.49 | 8.24 |
| *psb*A-*trn*K | LSC | IGS | 0 | NT | NT | 1.23 | 0 | NT | NT | 1 | 11.32 | 91.95 | 9.88 |
| *trn*K-*rps*16 | LSC | IGS | - | 0 | 0 | 1.55 | - | 0 | 0 | 1 | - | 105.19 | 16.67 |
| *mat*K Exon | LSC | Exon | 0 | NT | NT | 0.44 | 0 | NT | NT | 1 | 20.54 | 85.83 | 11.07 |
| *rps*16-*trn*Q | LSC | IGS | - | 0 | NT | 0.93 | - | 0 | NT | 2 | - | 127.23 | 9.88 |
| *rps*16 Intron | LSC | Intron | - | 0 | 1.32 | 0.42 | - | 0 | 1 | 1 | - | 51.83 | 12.08 |
| *trn*Q-*psb*K | LSC | IGS | 0 | 0 | NT | 1.82 | 0 | 0 | NT | 2 | 21.82 | 57.55 | 13.94 |
| *psb*K-*trn*S | LSC | IGS + Gene | 0 |  | 0 | 2.38 | 1 | 0 | 1 | 2 | 25.32 | 48.39 | 24.76 |
| *trn*S-*trn*G | LSC | IGS | 0 | NT | NT | - | 0 | NT | NT | - | 26.87 | - | - |
| *trn*G Intron | LSC | Intron | 0 | 0 | NT | 2.90 | 0 | 0 | NT | 0 | 21.65 | - | 14.49 |
| *trn*G-*atp*A | LSC | IGS + Gene | 0 | - | - | 0 | 0 | - | - | 0 | 9.17 | - | 12.38 |
| *atp*A Exon | LSC | Exon | 0 | - | - | 0.33 | 0 | - | - | 0 | 9.49 | - | 6.67 |
| *atp*F-*atp*H | LSC | IGS | 0 | NT | NT | 2.15 | 0 | NT | NT | 0 | 14.79 | 57.69 | 10.32 |
| *atp*F Intron/Exon | LSC | Intron + Exon | - | 0 | NT | - | - | 0 | NT | - | - | 63.14 | - |
| *atp*H-*atp*I | LSC | IGS | 0 | NT | NT | 0.39 | 0 | NT | NT | 4 | 24.05 | 108.20 | 14.90 |
| *atp*I Exon | LSC | Exon | 0 | NT | NT | 0.67 | 0 | NT | NT | 0 | 8.35 | 34.94 | 2.67 |
| *atp*I-*rps*2 | LSC | IGS | 0 | NT | NT | 0 | 0 | NT | NT | 0 | 10.08 | 44.94 | 8.48 |
| *rps*2 Exon | LSC | Exon | 0 | NT | NT | - | 0 | NT | NT | - | 11.44 | 29.36 | - |
| *rps*2-*rpo*C2 | LSC | IGS | 0 | 0 | 0 | 0.95 | 0 | 0 | 0 | 1 | 9.57 | 56.87 | 7.62 |
| *rpo*C2-*rpo*C1 | LSC | IGS | 0 | 0 | 0 | 0.43 | 0 | 0 | 0 | 1 | 18.47 | 55.76 | 11.97 |
| *rpo*C1 Intron/Exon1 | LSC | Intron + Exon | 0 | 0 | 0 | 0.30 | 0 | 0 | 0 | 3 | 12.37 | 38.74 | 8.33 |
| *rpo*C1 Exon2 | LSC | Exon | 0 | NT | NT | 0.47 | 0 | NT | NT | 0 | 9.78 | 39.16 | 6.06 |
| *rpo*B-*trn*C | LSC | IGS | 0 | 0 | NT | 1.00 | 0 | 0 | NT | 3 | 25.66 | 88.89 | 14.04 |
| *trn*C-*pet*N | LSC | IGS | 0 | 0 | 0 | 0 | 0 | 0 | 0 | 2 | 27.27 | - | 8.96 |
| *pet*N-*trn*D | LSC | IGS + Gene | 0 | NT | NT | - | 1 | NT | NT | - | 17.78 | 101.39 | - |
| *trn*D-*trn*T | LSC | IGS + Gene | 0.79 | - | - | 0.77 | 1 | - | - | 1 | 21.53 | - | 11.49 |
| *trn*T-*psb*D | LSC | IGS | 0 | - | NT | 1.21 | 0 | - | NT | 4 | 37.28 | - | 5.45 |
| *psb*D Exon | LSC | Exon | 1.30 | NT | NT | 0 | 0 | NT | NT | 0 | 12.59 | 50.23 | 4.17 |
| *psb*C-*psb*Z | LSC | IGS + Gene | 0 | 0 | 0 | 0.39 | 0 | 0 | 0 | 0 | 10.19 | 73.67 | 6.59 |
| *psb*C Exon | LSC | Exon | 0 | NT | NT | 0.27 | 0 | NT | NT | 0 | 8.57 | 46.18 | 5.50 |
| *psb*Z-*trn*fM | LSC | IGS + Gene | 0 | - | - | 0.48 | 0 | - | - | 4 | 10.25 | 111.73 | 5.71 |
| *trnf*M-*psa*B | LSC | IGS + Gene | 0 | - | NT | 0.51 | 1 | - | NT | 1 | 16.29 | - | 4.10 |
| *psa*A-*ycf*3 | LSC | IGS | 0 | 0 | 0 | 0.41 | 0 | 0 | 0 | 0 | 18.77 | 67.00 | 9.82 |
| *ycf*3 Intron2 | LSC | Intron | 1.47 | 0 | 1.55 | 0.45 | 0 | 0 | 0 | 2 | 12.77 | 50.06 | 5.41 |
| *ycf*3 Intron1 | LSC | Intron + Exon | 2.37 | NT | NT | 0.39 | 0 | NT | NT | 0 | 11.85 | 51.45 | 6.20 |
| *ycf*3-*rps*4 | LSC | IGS + Gene | 0 | 0 | NT | 0 | 0 | 0 | NT | 1 | 16.93 | 71.76 | 7.96 |
| *trn*L Intron | LSC | Intron | 0 | 0 | 0 | 0 | 0 | 0 | 0 | 1 | 12.15 | 45.83 | 4.00 |
| *trn*L-*ndh*J | LSC | IGS + Gene | 0 | NT | NT | 0.29 | 0 | NT | NT | 3 | 18.56 | 100.00 | 14.49 |
| *ndh*C-*trn*V | LSC | IGS | 0.81 | NT | NT | - | 0 | NT | NT | - | 13.57 | 89.17 | - |
| *trn*V Intron | LSC | Intron | 0 | 0 | NT | 0 | 0 | 0 | NT | 0 | 13.28 | 70.33 | 6.90 |
| *trn*V-*atp*B | LSC | IGS + Gene | 0 | 0 | 0 | 0.80 | 0 | 0 | 0 | 2 | 15.02 | 42.86 | 9.64 |
| *atp*B Exon | LSC | Exon | 0 | NT | NT | 1.13 | 0 | NT | NT | 0 | 7.81 | 41.40 | 3.39 |
| *atp*B-*rbc*L | LSC | IGS | 0 | 0 | 0 | 0 | 0 | 0 | 2 | 0 | 14.44 | 74.57 | 5.67 |
| *rbc*L Exon | LSC | Exon | 0 | NT | NT | 0.85 | 0 | NT | NT | 0 | 15.72 | 39.90 | 10.17 |
| *rbc*L-*acc*D | LSC | IGS | 0 | - | - | 1.00 | 0 | - | - | 2 | 16.77 | - | 13.53 |
| *acc*D-*psa*I | LSC | IGS | 0 | - | - | - | 0 | - | - | - | 3.92 | - | - |
| *acc*D exon | LSC | Exon | 0 | - | - | 0 | 0 | - | - | 0 | 9.57 | - | 12.39 |
| *psa*I-*ycf*4 | LSC | IGS | 0 | 0 | 2.48 | 0 | 0 | 0 | 1 | 0 | 2.93 | 93.67 | 13.33 |
| *ycf*4 Exon | LSC | Exon | 0 | NT | NT | 0.78 | 0 | NT | NT | 0 | 6.93 | 32.33 | 12.40 |
| *ycf*4-*pet*A | LSC | IGS | - | 0 | NT | - | - | 0 | NT | - | - | 46.52 | - |
| *pet*A-*psb*L | LSC | IGS + Gene | - | - | NT | 0 | - | - | NT | - | - | 93.37 | 7.35 |
| *pet*A Exon | LSC | Exon | 0 | NT | NT | 0 | 0 | NT | NT | 0 | 12.00 | 36.76 | 8.55 |
| *pet*L-*trn*P | LSC | IGS + Gene | 0 | 0 | 0 | 0.95 | 0 | 0 | 0 | 0 | 18.87 | 48.48 | 6.67 |
| *trn*P-*rps*18 | LSC | IGS + Gene | 0 | 0 | 1.70 | - | 0 | 0 | 1 | - | 10.28 | 125.00 | - |
| *rps*18-*rps*12 | LSC | IGS + Gene | 0.86 | 0 | NT | 0.24 | 0 | 0 | NT | 2 | 14.27 | 50.34 | 6.19 |
| *rps*12-*clp*P | LSC | IGS | 0 | NT | NT | - | 1 | NT | NT | - | 11.95 | - | - |
| *clp*P Intron2 | LSC | Intron | 0 | - | NT | 0 | 1 | - | NT | 0 | 13.63 | - | 9.52 |
| *clp*P Intron1 | LSC | Intron + Exon | 0 | - | - | 0 | 0 | - | - | 4 | 16.27 | - | 9.43 |
| *rps*12-*psb*B | LSC | IGS + Gene | - | 0 | 0 | - | - | 0 | 0 | - | - | 40.26 | - |
| *psb*B Exon | LSC | Exon | 0.75 | - | NT | 0.25 | 0 | - | NT | 0 | 6.24 | 29.75 | 3.43 |
| *clp*P-*psb*B | LSC | IGS | 0 | NT | NT | 0.21 | 0 | NT | NT | 4 | 17.26 | - | 9.58 |
| *pet*B Intron/Exon2 | LSC | Intron + Exon | 0 | 0 | NT | 0.26 | 0 | 0 | NT | 0 | 14.53 | 61.62 | 5.13 |
| *pet*B-*pe*tD | LSC | IGS | - | 0 | 0 | 0.35 | - | 0 | 0 | 1 | - | 65.31 | 7.72 |
| *pet*D Intron/Exon2 | LSC | Intron + Exon | 0 | 0 | NT | 0.65 | 0 | 0 | NT | 0 | 14.97 | 55.66 | 5.88 |
| *pet*D-*rpo*A | LSC | IGS | 0 | 0 | 0 | 1.96 | 0 | 0 | 0 | 2 | 15.68 | 62.50 | 11.76 |
| *rps*11-*rps*8 | LSC | IGS + Gene | 0 | NT | NT | 0.33 | 0 | NT | NT | 0 | 11.34 | - | 7.84 |
| *rps*8-*rpl*16 | LSC | IGS + Gene | 0.76 | 0 | NT | - | 1 | 0 | NT | - | 9.41 | 50.64 | - |
| *rpl*16 Intron | LSC | Intron | 1.05 | NT | - | - | 0 | NT | NT | - | 17.53 | 88.26 | - |
| *rpl*16-*rps*3 | LSC | IGS | 1.44 | 0 | 0 | 0 | 1 | 0 | 0 | 1 | 6.73 | 60.72 | 10.39 |
| *rps*3 Exon | LSC | Exon | 0 | NT | NT | 0 | 0 | NT | NT | 0 | 6.38 | 51.23 | 13.89 |
| *rpl*22-*rpl*2 | LSC | IGS + Gene | 0 | 0 | 0 | - | 0 | 0 | 0 | - | 7.32 | 34.22 | - |
| *rpl*2 Intron/Exon1-2 | IR | Intron + Exon | 0 | NT | NT | - | 0 | NT | NT | - | 2.69 | 10.05 | - |
| *rpl*23-*ycf*2 | IR | IGS + Gene | 0 | NT | NT | 0 | 0 | NT | NT | 0 | 0 | - | 1.17 |
| *ycf*2-*ndh*B | IR | IGS + Gene | 0 | NT | - | - | 0 | NT | - | - | 2.73 | - | - |
| *ndh*B Exon2 | IR | Exon | 0 | NT | NT | 0 | 0 | NT | NT | 0 | 1.41 | 5.24 | 0.95 |
| *ndh*B Intron/Exon1 | IR | Intron + Exon | 0 | 0 | NT | 0.77 | 0 | 0 | NT | 0 | 1.51 | 5.73 | 1.03 |
| *ndh*B-*rps*7 | IR | IGS | 0 | 0 | 0 | 0 | 0 | 0 | 0 | 0 | 1.78 | 13.56 | 0 |
| *rps*12-*trn*V | IR | IGS | 0 | 0 | NT | - | 0 | 0 | NT | - | 0.78 | 12.47 | - |
| *rps*12 Intron/Exon | IR | Intron + Exon | 0 | 0 | 0 | 0 | 0 | 0 | 0 | 0 | 0 | 11.53 | 0 |
| *trn*V-*rrn*16 | IR | IGS | 0 | NT | NT | 0 | 0 | NT | NT | 0 | 3.86 | 11.45 | 0 |
| *rrn*16 Exon | IR | Exon | 0 | NT | NT | 0 | 0 | NT | NT | 0 | 0.79 | 2.81 | 0 |
| *rrn*16-*trn*I | IR | IGS | 0 | 0 | 0 | 0 | 0 | 0 | 0 | 0 | 9.38 | 3.06 | 1.85 |
| *trn*I Intron | IR | Intron | 0 | 0 | 0 | 0 | 0 | 0 | 0 | 0 | 1.23 | 15.71 | 0.73 |
| *trn*A-*rrn*23 | IR | IGS | 0 | - | NT | - | 0 | - | NT | - | 0 | 19.70 | - |
| *trn*A Intron | IR | Intron | 0 | 0 | NT | 0 | 0 | 0 | NT | 0 | 2.72 | 14.76 | 0.89 |
| *rrn*4.5-*trn*N | IR | IGS + Gene | 0.85 | 0 | 0 | 1.03 | 0 | 0 | 0 | 0 | 2.84 | 18.96 | 9.23 |
| *rps*15-*ycf*1 | SSC | IGS | 0 | - | - | 1.56 | 0 | - | - | 3 | 35.32 | - | 18.75 |
| *ycf*1-*rrn*5 Dio | SSC | IGS + Gene | 0.80 | NT | NT | - | 0 | NT | NT | - | 4.26 | - | - |
| *ycf*1-*rrn*5 | SSC | IGS + Gene | - | NT | - | 0.51 | - | NT | - | 1 | - | - | 4.62 |
| *ndh*A Intron | SSC | Intron + Exon | 0.90 | 0.93 | NT | 2.45 | 0 | 0 | NT | 0 | 16.69 | 78.80 | 3.00 |
| *ndh*H Exon | SSC | Exon | 1.13 | NT | NT | 1.11 | 0 | NT | NT | 0 | 12.76 | 52.52 | 6.67 |
| *ndh*G-*ndh*I | SSC | IGS | 0.97 | NT | NT | 0.60 | 0 | NT | NT | 3 | 10.99 | 51.79 | 14.88 |
| *ndh*G Exon | SSC | Exon | 0 | NT | - | - | 0 | NT | - | - | 8.85 | 50.31 | - |
| *psa*C-*ndh*G | SSC | IGS + Gene | 0 | NT | NT | - | 0 | NT | NT | - | 15.22 | 49.62 | - |
| *ccs*A-*ndh*D | SSC | IGS | 1.36 | 0 | 0 | - | 0 | 0 | 0 | - | 19.91 | 72.73 | - |
| *ndh*D Exon | SSC | Exon | 0 | NT | NT | 0 | 0 | NT | NT | 0 | 6.00 | 53.02 | 6.13 |
| *ccs*A Exon | SSC | Exon | 1.25 | NT | NT | 1.25 | 0 | NT | NT | 0 | 17.15 | 50.59 | 16.67 |
| *rpl*32-*ccs*A | SSC | IGS + Gene | 0 | 0 | 0 | - | 0 | 0 | 0 | - | 26.22 | 92.32 | - |
| *ndh*F-*rpl*32 | SSC | IGS | 2.65 | NT | NT | - | 1 | NT | NT | - | 92.58 | 118.18 | - |
| Mean | LSC |  | 0.18 | 0.00 | 0.34 | 0.59 | 0.13 | 0.00 | 0.29 | 1.02 | 14.26 | 62.82 | 9.07 |
| Mean | IR |  | 0.06 | 0.00 | 0.00 | 0.16 | 0.00 | 0.13 | 0.20 | 0.00 | 2.11 | 11.16 | 1.44 |
| Mean | SSC |  | 0.75 | 0.31 | 0.00 | 1.07 | 0.09 | 0.00 | 0.00 | 1.00 | 23.37 | 68.84 | 10.10 |
| Mean | All |  | 0.24 | 0.10 | 0.11 | 0.57 | 0.07 | 0.04 | 0.18 | 0.87 | 13.25 | 47.61 | 8.07 |
